# Draft genome sequence of a marine coccolithophore *Chrysotila carterae* NIES-4509

**DOI:** 10.1128/mra.01357-24

**Published:** 2025-06-09

**Authors:** Kohei Yoneda, Shigekatsu Suzuki, Masanobu Kawachi, Hirotoshi Endo, Akihiro Kato, Tomoya Takeda, Yoshiaki Maeda, Iwane Suzuki

**Affiliations:** 1 Institute of Life and Environmental Sciences, University of Tsukuba 13121, Tsukuba, Japan; 2 Biodiversity Division, Microbial Culture Collection of National Institute for Environmental Studies, Tsukuba, Japan; 3 National Institute of Technology Tsuruoka College 13299, Tsuruoka, Japan; 4 Environment and Materials Development Department, Technology Development Division, Seiko Epson Corp, Suwa, Japan; University of California Riverside, Riverside, California, USA

**Keywords:** haptophytes, coccolith, *Chrysotila*

## Abstract

The present study reports the draft genome assembly of a transformable marine coccolithophore *Chrysotila carterae* NIES-4509. The draft genome consisted of approximately 451.7 Mbp, with 57.9 % G + C content and 9,265 contigs. This assembly is expected to be utilized for understanding the cellular physiology of marine haptophytes.

## ANNOUNCEMENT

A group of haptophytes, coccolithophores, has calcareous plates (coccoliths) around cells, and they play an important role in the carbon circulation of the marine environment (1). To date, the genome of *Emiliania huxleyi* has been sequenced and is used as a model species in coccolithophores (2). However, the difficulty in the genetic transformation of *E. huxleyi* hinders molecular characterization of coccolith formation. A coccolith-forming marine haptophyte *Chrysotila*, formerly known as *Pleurochrysis* (3), is a transformable haptophyte (4). Recently, the genome of *C. roscoffensis* NMBjih026-8 was reported (5); however, this strain and its genome data are officially unavailable. To provide genomic information for genetic transformation and elucidate the cellular physiology, including coccolith formation, we sequenced the draft genome of *C. carterae* NIES-4509.

*Chrysotila carterae* NIES-4509 was acquired from the National Institute for Environmental Studies, Japan, and statically cultivated in modified MA-ESM (6) at 20°C under a 14 h:10 h light-dark cycle (approx. 70 µmol photons m^−2^ sec^−1^) for 5 days. The extracted genomic DNA using Genomic-tip 20 /G (QIAGEN N.V., Hilden, Germany) was fragmented to approximately 10 to 20 kb using g-TUBE (Covaris LLC., Woburn, MA, USA). The library that was constructed using SMRTbell gDNA Sample Amplification Kit (PacBio, Menlo Park, CA, USA) and SMRTbell Express Template Prep Kit 2.0 (PacBio) was sequenced using Revio Polymerase kit (PacBio) and Revio (PacBio). The overhang adopter sequence was removed using SMRT Link ver. 13.0.0.207600. The obtained sub-reads were aligned to generate consensus sequences, and then, the high-fidelity reads (>Q20) were selected from the generated consensus sequences. Lima ver.2.7.1 (7) and pbmarkdup ver.1.0.3 (8) were, respectively, used for the elimination of PCR adaptor sequences and duplicate PCR reads. Short high-fidelity sequences (<1,000 bp) were removed using Filtlong ver.0.2.1 (9). Finally, we acquired 4,506,725 reads (22.6 Gbp) and assembled them using Flye ver.2.9.4 (10) with a pacbio-hifi option. Haplotigs were purged using Purge_haplotigs ver.1.1.3 (11), and putative organelle genomes, which were detected by Whokaryote ver.1.1.2 (12), were removed. Repeat sequences were predicted and masked using RepeatModeler ver.2.0.5 (13) and RepeatMasker ver.4.1.5 (14). Gene prediction and functional annotation were performed using the Funannotate pipeline ver.1.8.15 (15) with RNA-seq reads (SRR1300347–SRR1300349).

The total size of the draft genome assembly was approximately 451.7 Mbp with 50.0× coverage to the haploid assembly size, 57.9% G + C content, and 9,265 contigs (Table 1). The contig N_50_ value was 76.3 kbp, and 47,979 protein-coding genes were predicted. BUSCO analysis based on the genome data with the eukaryota data set (ver. 5.7.1) showed 48.6% complete and 20.0% fragmented BUSCOs. Compared with the *C. roscoffensis* genome (636 Mbp in size) with 23,341 protein-coding genes (5), almost twice the number of protein-coding genes was predicted in the *C. carterae* genome. The *C. roscoffensis* genome has a complex gene structure with a relatively high density of long introns, suggesting that the gene number of *C. carterae* might be overestimated due to genome fragmentation. Indeed, the BUSCO analysis using the protein data set showed many fragmented BUSCOs. The *C. carterae* genomic data should be useful information for genetic transformation in future research.

**TABLE 1:** Summary data of PacBio sequencing analysis and features of the draft genome of *Chrysotila carterae* NIES-4509 in the present study

Parameters	Values
PacBio sequencing	
Total number of reads	4,506,725
Average size of reads (bp)	5,014
Total number of sequenced nucleotides (bp)	22,597,708,470
Genome assembly	
Total genome size (bp)	451,748,466
Number of contigs	9,265
G + C content (%)	57.9
Genome coverage (haploid)	50.0
Repeat sequence (%)	67.7
Number of protein-coding genes	47,979
Genome completeness by BUSCO (%]	
Genome data set	Complete BUSCOs: 48.6, Fragmented BUSCOs: 20.0, Missing BUSCOs: 31.4
Protein data set	Complete BUSCOs: 29.4, Fragmented BUSCOs: 27.5, Missing BUSCOs: 43.1

## Data Availability

The draft genome sequence of C. carterae NIES-4509 was deposited in DDBJ under accession number BAAGDD010000001-BAAGDD010009265 (BioProject number PRJDB19668). PacBio reads are available in DDBJ Sequence Read Archive (DRA) under DRA Run accession number DRR622298. GFF, CDS, and protein sequence file are deposited to figshare (DOI: 10.6084 /m9.figshare.28862195).
